# Laser Interstitial Thermal Therapy for Cavernous Malformations: A Systematic Review

**DOI:** 10.3389/fsurg.2022.887329

**Published:** 2022-05-13

**Authors:** Omid Yousefi, Mohammadmahdi Sabahi, James Malcolm, Badih Adada, Hamid Borghei-Razavi

**Affiliations:** ^1^ Student Research Committee, Shiraz University of Medical Sciences, Shiraz, Iran; ^2^ Neurosurgery Research Group (NRG), Student Research Committee, Hamadan University of Medical Sciences, Hamadan, Iran; ^3^ Department of Neurosurgery, Emory University, Atlanta, GA, United States; ^4^ Department of Neurological Surgery, Pauline Braathen Neurological Center, Cleveland Clinic Florida, Weston, FL, United States

**Keywords:** laser interstitial thermal therapy, LITT, cavernous malformation, neurosurgery, systematic review

## Abstract

**Background:**

Microsurgical resection of intracranial cavernous malformations (CM) is regarded as the standard treatment, but in recent years, there has been a trend toward minimally invasive procedures like ablation of such lesions by using laser interstitial thermal therapy (LITT).

**Methods:**

A systematic search using keywords ‘laser interstitial thermal therapy’ OR ‘LITT’ AND ‘cavernoma’ OR ‘cavernous angiomas’ OR ‘cavernous malformations’ was conducted in MEDLINE (PubMed), Scopus, Embase, and Cochrane electronic bibliographic databases and studies reporting the outcome of LITT procedure on intracranial CM were included. The demographic data, symptoms of patients, location and size of the lesion, and surgical outcome were extracted from the articles.

**Result:**

Six studies, reporting the outcome of 33 patients were included in this review. In 26 patients, CM was identified as the epileptogenic foci and in others, CM was the source of headache or focal neurological deficits. LITT led to a satisfactory outcome in all patients except for three who achieved improvement in symptoms after the open resection of the lesion. Most of the post-operative complications were transient and resolved at the time of the last follow up. Cyst formation at the previous ablated CM site was reported as the long-term complication of LITT in one case.

**Conclusion:**

LITT can provide a comparable outcome to the open resection of CMs, by having less invasiveness, even in deep and eloquent area lesions, and complications that are often temporary and disappear gradually. However, technical issues, such as thermal monitoring during the procedure, are considered a challenge for this procedure in CMs. Further studies with a larger population are needed to report this method's long-term outcome and complications on CMs.

## Introduction

Cavernous malformations (CMs) are clumps of endothelium-lined multilobulated arteries that lack brain parenchyma and have a ‘popcorn’ or ‘mulberry’ look (1–3). They are accounted as the most common intracranial vascular malformations, having an incidence of 0.1%–0.8%. Probable risk of hemorrhage (overall up to 2.4% per year), seizure, and focal neurological deficits (FND) represent the main concerns regarding CMs (4, 5). A meta-analysis of individual patient data on the clinical course of untreated cerebral CMs demonstrated an estimated 5-year risk of intracranial hemorrhage (ICH) is 15.8% (6), while the 5-year risk of a first hemorrhage was lower than the risk of recurrent hemorrhage (7). Another study showed that the overall cumulative 5-year risk of re-hemorrhage was 24.1% (8). These data indicate the priority of therapeutic intervention in patients with cerebral CMs. Microsurgical resection of symptomatic CMs during the subacute phase of hemorrhage is regarded as the goal standard treatment in the management of CMs (2, 9).

The use of 50 to 90 degrees Celsius heat to ablate cerebral lesions has been the topic of numerous studies over the last 30 years (10, 11). Magnetic resonance-guided laser interstitial thermal therapy (LITT) has been used in the minimally invasive surgical treatment of different pathologies such as tumors, seizure foci, metastasis, etc. (10, 12, 13) Outcome of this procedure is comparable to the open surgery, and these findings promise a potential therapeutic modality for some types of brain lesions. In some conditions like hypothalamic hamartoma, LITT is suggested to be regarded as the first-line treatment option (14). However, thermal management and monitoring have remained the main challenges in avoiding injury to the adjacent structures and post-operative complications.

During the last years, there have been reports of the application of LITT for intracranial CMs. In this study, we aim to systematically review the literature, reflecting outcomes and complications of the treatment of CMs by using LITT.

## Materials and Method

This systematic review was conducted according to Preferred Reporting Items for Systematic Reviews and Meta-Analyses (PRISMA) criteria using the following study question: Is laser interstitial thermal therapy (LITT) safe and effective for cavernomas? (15, 16).

### Literature Search and Selection Criteria

We reviewed published articles between 2015 and 2021 in English and with no date restrictions. The following databases were explored to find reports on the safety and efficacy of LITT for cavernous malformations: MEDLINE (using PubMed), Scopus, Embase, and Cochrane library. The keywords and terms used in this study include ‘Laser Interstitial Thermal Therapy’ OR ‘LITT’ OR ‘Laser ablation’ AND ‘cavernous malformation’ OR ‘cavernoma’ OR ‘cavernous angioma’ OR ‘cavernous haemangioma’ OR ‘cerebral cavernous malformation’. The date of the last search was November 2021. Moreover, all relevant cited references in the original articles were searched to find articles which were not indexed by the databases mentioned above. The articles were reviewed by EndNote X7.1 (Thomson Reuters).

The final selection was made using the following inclusion criteria: (1) case series, and prospective and retrospective studies assessing LITT on cerebral cavernous malformation; and (2) studies that provided outcomes of LITT for cerebral cavernous malformation. Exclusion criteria consisted of (1) animal studies, letters to the editor, expert opinions, commentary, (2) studies that only delineated LITT on vascular malformations other than cerebral cavernous malformation, and (3) records whose patients have been discussed in other articles and there is overlap between patients.

**Figure 1:**
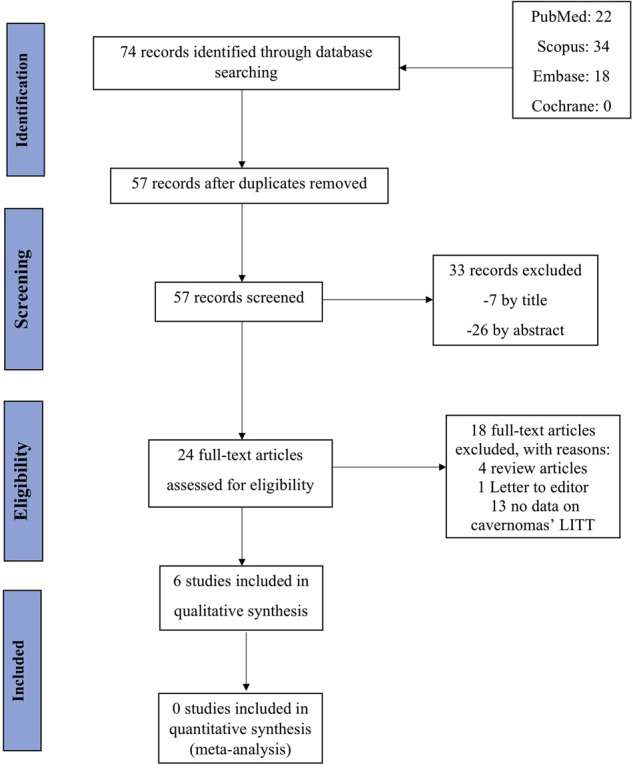
The PRIMSA diagram illustrates the search and selection process that we used to develop the overview.

This query identified 74 papers that were assessed for relevance by two independent reviewers (O.Y. and M.S.). The initial search identified 22 papers in MEDLINE (PubMed), 34 papers in Scopus, 18 papers in Embase, and 0 papers in Cochrane. After removing 17 duplicate papers, titles and abstracts of 57 records were screened, of which 33 records were irrelevant. This resulted in a final selection of 24 papers which were surveyed for eligibility. Out of 24 records, 5 articles were excluded including 4 review articles and 1 letter to the editor. Of the 19 remaining articles, 13 studies were removed as they did not include data on cavernomas that underwent LITT. As a result, 6 studies were included in this study; and the search strategy is summarized in Figure 1.

### Data Extraction

Data extraction was performed based on a predefined protocol by one author (O.Y.) and was rechecked by another one (M.S.). Disagreements were resolved by a third author (J.M.). The extracted data included: (1) patient demographics, including age and gender distribution; (2) region that vascular lesion was located and also the number of these lesions in any corresponding areas; (3) lesion size (4) patients’ symptoms before LITT; (5) intra-operative complications; (6) mean follow-up; (7) post-operative symptoms; (8) post-operative imaging; (9) post-surgical complications; and (10) re-operation. Any other data not relevant to the aim of this systematic review was ignored.

Since LITT for cavernomas is a rather uncommon entity, with the majority of data coming from small sample sizes and a lack of high-power studies, we included case reports and case series in the analysis.

## Results

### Study Characteristics

Reports of thirty-three different patients who underwent LITT for the ablation of intracranial CM since 2019 are included in the literatures. All patients’ brain MRI revealed an intracranial CM with a distinct ‘popcorn’ morphology, with a rim of hypointensity on T2-weighted sequences and a conspicuous blooming artifact on susceptibility-weighted sequences confirming hemosiderin presence. Most of the procedures took place in adult patients with a mean age of 34.8 years. The characteristics of the patients are illustrated in Table 1 (2, 4, 11, 17–19). Except for one patient, who sought early intervention to terminate antiepileptic medicines, the existence of a cerebral CM producing drug resistant seizures was an indication for surgery in Willie at al. study (4). Similarly, all patients in Satzer et al. survey had cerebral CM-related drug-resistant focal epilepsy (2).

**Table 1:** Characteristics and outcomes of the patients, who underwent LITT for cavernoma.

Study	Number of cases	Age	Regions & number	Lesion size	Symptoms	IOC	Mean F/u	post-op symptoms	Post-op imaging	Post op complications	re-operation
Gamboa et al. (17)	2	69	Brain stem	1.8 cm	horizontal diplopia, left facial numbness, paresthesia	-	18 m	resolved diplopia and left facial numbness with some residual left-sided weakness and ataxia.	small zone of injury in the posterior internal capsule and cerebral peduncle, circumferential edema around the CM, involuted pontine CM	some worsened left-sided facial numbness, left-sided weakness, and dysarthria that gradually improved	-
		46		1.6 cm	vertigo, diplopia (right-sided exotropia), dysarthria, left-sided weakness	–	12 m	improvement in diplopia, vertigo, dysarthria, and left-sided weakness	involution of the central pontine CM	–	–
Carminucci et al. (18)	1	59	Temporal	1 * 1 cm	seizure	none	30 m	Seizure free till 30 months when patients developed seizure	2 * 2 cyst in 30 months MRI	temporal growing cyst and re-operation	Due to growing cyst
Willie et al. (4)	19	40.3	Frontal (4) Parietal (1) Temporal (14)	0.7 ± 0.6 cm^3^	Seizure	Extended ablation (1)	30.6 ± 12.6 m	Engel Class: IA (10) IB (2) IC (1) ID (1) IID (1) IIIA (1) IVA (1) NA (2)	Mean 71% reduction in CCM size	Non disabling superior quadrantanopia (1) Hand weakness (recovered) (1)	2 cases
Satzer et al. (2)	6 patients 7 CCM	47	temporal (3) frontal (1) parietal (1) occipital (2)	0.7 cm^3^	seizure	none	24 m	Engle class: IA (4) IC (1) II (1)	24% reduction in size of hypointensity	blurry vision (1) single episode of seizure (1)	
Malcolm et al. (19)	4	27	Thalamus	0.6 cm^3^	headache		20 m	Improved	53.75% reduction in size	Mild transient paresthesia	
		41	Putamen	2.6 cm^3^	headache	Device malfunction and saline leakage into brain		Improved		Transient upper extremities apraxia	
		14	Thalamus	4.2 cm^3^	hemorrhage causing spastic hemiparesis and hemianopia			No recurrent hemorrhage		Persistent exacerbation of hemiparesis and hemianopia	
		62	subthalamus	0.92 cm^3^	Hemorrhage headache	asymptomatic hemorrhage in tract		No recurrent hemorrhage		–	
Lawrence et al. (11)	1	20	pons	2.4 cm × 2.6 cm	left-sided paresthesia, weakness, and gait imbalance	–	19 m	NR	1.3 cm × 1.2 cm	Diplopia improved over the time	–

### Follow-up

The mean follow-up duration was 21.3 months (12–42 months). The Follow-up sessions included both clinical evaluation and imaging assessment. Patients were asked either by phone or in-person follow-up appointment about seizures, side effects, and medication status (2, 4). The Engel classification method was used to record seizure outcomes (20). Patients who did not achieve seizure freedom after ablation alone were considered candidates for further surgical operations (4).

### Location and Presentation

As demonstrated in Table 1, 3 patients had CM in the brain stem region, 4 patients in the basal ganglia area, 18, 5, 2 and 2 patients in the temporal, frontal, parietal and occipital lobes, respectively. Five patients had a history of hemorrhage in the CM site, which caused FNDs. LITT led to improvement in preoperative symptoms in most patients, and no one has had to undergo a second surgery. None of the patients with a previous history of hemorrhage developed rebleeding at the CM site during follow up.

The seizure was the most common pre-operative symptom observed in 26 patients who had CM in different brain lobes. After LITT for 24 patients who had seizure, with excluding 2 patients whose data were not available, based on Engel classification (20), 20 patients (83.3%) achieved class I (seizure free) and 4 patients (16.7%) were categorized as higher classes. Among seizure free patients, 15 patients (62.5%) achieved excellent seizure control (IA), while 2 (8.3%), 2 (8.3%) and 1 (4.2%) patient were IB, IC and ID, respectively. In the report of Carminucci et al., a patient who had post-stereotactic radiosurgery (SRS) temporal CM formation had a stable control of the seizure till 30 months, when the growing cyst formation at the location of ablation was recognized as the cause of the new-onset seizure attacks (18). In the series of Willie et al. 2 cases with a history of impaired awareness, underwent the second surgery after stereotactic electroencephalography and precise identification of the seizure foci (4).

### Imaging

Various applications, such as OsiriX MD (Pixmeo SARL, Geneva, Switzerland) (4), Visage 7 (Visage Imaging, San Diego, CA, USA) (2), Horos 3.3.6 (Purview, Annapolis, Maryland, USA) (19), were used in different studies to provide CMs dimensions and volumetric analyses. Almost all the cases had involution and reduction in the size of the CM in the follow-up imaging and interval imaging and pathologic examination suggest that LITT leads to involution of intracranial CMs. According to studies that compared imaging before surgery and at the last follow-up, the average lesion size decreased by roughly 59 percent. Perilesional edema was also a common finding in immediate post-operative imaging.

### Intra-Operative Complications

Intra-operative complications (IOC) occurred in three different cases: (1) Extended ablation into the temporal lobe resulting in a non-disabling visual field defect (4), (2) Device malfunction, and coolant leakage into the brain causing incomplete ablation (19), (3) asymptomatic hemorrhage along the trajectory tract (19).

### Post-Operative Complications

Most of the time, the post-operative complications were transient and gradually resolved. The long-term complication of LITT was only reported in the Carminucci et al. study, and they reported a cyst that formed after LITT (18).

## Discussion

CMs, by having an incidence of 0.1%–0.8% in the normal population, are the most common intracranial vascular malformation and are seen in the familial form in 40 to 60% of cases (1, 5). They can cause headache, FND and seizure and have a potential risk of hemorrhage. CMs are mostly located in supratentorial regions, but infratentorial CMs, representing 1/5 of lesions, have a higher risk of bleeding (up to 10.6% per year) and, due to their adjacency to sensitive structures, cause more severe FNDs (5).

Although conservative treatment along with annual imaging are the proposed policy in cases with asymptomatic CMs, the standard treatment in symptomatic patients is the surgical resection. The initial reports of surgical removal of this pathology return to more than a hundred years ago, and during the years, the efficacy and safety of surgical approaches have been improved by advancements in technics and instruments (21).

During the last decades, there has been a trend toward the application of minimally invasive methods, which provide satisfactory outcomes and a low rate of complications. SRS has been used for different pathologies, but the procedure’s efficacy is not predictable in all cases (2). For instance, it is observed that control of epilepsy is achieved lately after SRS and its efficacy in complex seizure situations is under debate (4). Risk of secondary cyst and CM formation should also be considered (2, 18).

Since the early 90s, there have been reports of thermal ablation of cerebral lesions. Since the initial days of using this method, the main challenges were avoiding thermal injury to the adjacent structures and possible deviation (22). Tendency of the lesions for bleeding and providing a safe trajectory are other barriers.

MRI-guided LITT provides a real time (or near to real-time) monitoring of thermal changes of the tissue by obtaining different sequences such as T1 weighted, water proton resonance frequency, etc. Outcomes of the LITT for other pathologies such as tumors, metastasis, seizure foci, etc., have been discussed in numerous reports and reviews (13).

In certain pathologies, such as seizure, LITT's results are comparable to those of open microsurgical techniques. According to our review of the limited available data, 83.3% of patients who underwent LITT for their CMs became seizure-free. A larger systematic review of 1226 patients who had supratentorial cerebral CMs who presented with seizure episodes and underwent microsurgical lesion removal resulted in seizure freedom in 75% of patients (23). Long-term follow-up after surgical resection of supratentorial CMs in Kwon et al. study demonstrated 82.1% of patients were free from impairing seizures. Kapadia et al. survey on patients with supratentorial CM after early surgery either by open craniotomy or microsurgical resection demonstrated the rate of seizure freedom at 1 year was 94.7% and 62.5% in patients with ≤2 and >2 seizures, respectively (24). Considering all data together, it seems that LITT should be considered a potent therapeutic modality in patients with CMs but in order to verify the efficacy of this treatment, longer follow-up in patients who received LITT is crucial.

During the last three years, the application of LITT for cerebral CMs has been in the spotlight. CMs are angiographically occult and have low flow blood circulation (5). These characteristics rationalize their low tendency for bleeding during the ablation or insertion of the probe into the CM (1, 19).

The majority of CMs are found in supratentorial regions, and up to 70% of individuals with such lesions have seizures (1). Ring of hemosiderin deposits, irritation, and gliosis of the brain parenchyma are regarded as the cause of the seizure (1, 3). Open microsurgical resection of CMs recognized as the epileptic foci can result in satisfactory outcomes in up to 80% of cases (21). Nevertheless, microsurgery for cerebral CMs necessitates direct access to the lesions by incision and craniotomy, which increases the potential of inadvertent collateral damage (particularly in deep or eloquent areas) (25), and lobectomies result in more severe neurocognitive impairments (26, 27).

SRS is a non-invasive option that results in seizure freedom rates, comparable to surgical resection. The seizure control rate by SRS for cerebral CMs has risen from 53% in studies two decades ago (28) to more than 80% in more recent studies (29). SRS for cerebral CM is linked to a prolonged temporal course and symptomatic radiation necrosis, particularly at doses used to treat seizures (25, 30, 31).

LITT, on the other hand, is minimally invasive and, in most situations, instantly effective. Furthermore, LITT's high rate of seizure control is equivalent to that of open surgery, and earlier ablation showed to be no obstacle to successful open surgery in those patients who were not initially seizure-free. Prior therapies, including as SRS and vagus nerve stimulation, were not contraindication for new ablative process (4).

It is also believed that early intervention after initial epileptic attacks can provide better results (1).

It is an essential factor to have a precise evaluation of seizure foci, especially in cases having multiple CMs and to have an intervention on the specified area, like what Satzer et al. performed for a patient with a familial type of CM (2). Expanded epileptogenic foci, like 2 cases in Willie et al. series who underwent open resection, is regarded as the cause of insufficient response of seizure to LITT (4, 10). An initial minimally invasive approach does not preclude later open resection.

It is assumed that removal of the hemosiderin ring is a crucial step in the control of the seizure, and in all LITT reports, ablation of the surrounding hemosiderin depositions was noted (1).

CMs have high susceptibility for thermal conduction, and the surrounding rim also enhances this event. Willie et al. observed that thermal spread around the CM can interrupt the precise thermal monitoring during LITT (32).

CMs which are located in either brain stem or basal ganglia are mostly presented with FNDs, and their surgical resection is always considered a challenge which requires experienced surgical teams. Resection of basal ganglia CMs have a 10% risk of morbidity, and neurologic deficits might be expected after the surgery, especially for lesions located in the globus pallidus and posterior limb of the internal capsule (3). Contralateral partial hemiparesis is the most common adverse event in the surgical treatment of basal ganglia CMs (33). Exacerbation of hemiparesis was also reported in a case of Malcolm et al., and it was among the only few adverse events of LITT for CMs which was not resolved over the time (19).

For CMs located in the brain stem, selecting the proper trajectory is an influencing factor on the resulted complications (17). Of the three patients with brain stem CM who underwent LITT, two developed transient post-operative FND which gradually improved, and it was attributed to the damaged structures along the trajectory (11, 17). In a study by Ashraf et al., performed on the posterior fossa neoplasms, a higher rate of complications was observed during and after the procedure for brain stem lesions compared to lesions in the cerebellum (34).

In most reports, postoperative adverse events were transient and were resolved or improved after a while (35). Some authors recommend performing LITT for lesions in sensitive regions in awake situation to monitor any change in neurological status, but some studies disagree due to the possibility movements and disruption of MRI based monitoring (19).

It is supposed that edema around the ablated target is the leading cause of transient subsequent adverse events (35). It is believed that after the ablation, it takes time (up to 90 days) that the size of the lesion would turn to half of the primary CM volume, and the perilesional change in the vasculature is assumed as the reason (12). Malignant edema and progressive FND were the cause of mortality in the Patel et al. study (35). However, LITT does not lead to mortality in reported CMs cases (35). A meta-analysis revealed that the total risk ratio of bleeding after SRS for brain stem CMs was 0.161 and 11.8% of patients experienced transitory or persistent neurological impairments (36).

In cases where surgeons doubt the sufficiency of the volume of the ablated target, using different trajectories is recommended, but it may increase the risk of complications (4, 35, 37).

In a study on 242 patients with brain stem CM, preoperative annual hemorrhage and re-hemorrhage rates were determined at 5.0% and 60.9% preoperatively, respectively, while the postoperative annual hemorrhage rate was 0.4 percent (38). In addition, about 40% of cavernoma remnants after surgery carry a risk of rebleeding (5), while re-hemorrhage was not seen after the LITT for CMs, even in cases with prior history of bleeding.

The findings of Monaco et al. study suggest that SRS may have a role in the treatment of symptomatic CMs of the brainstem, and appears to lower rebleeding rates from 32.38% to 8.22 within the first 2 years of follow-up (39).

In comparison with other therapeutic modalities such as SRS and surgery, LITT seems also a safe and compelling technique in the treatment of supratentorial CMs, with low risk of bleeding and FND, while for the purpose of confirming the safety and efficacy of this treatment, the longer follow-up and larger sample sizes are essential. Similar to brainstem CM’ surgical resection, which have higher risks and complications than other parts of the brain, LITT should be used with caution in these locations, and further experiments on larger cohorts are required to determine the method's safety and efficacy.

Pathological study of the resected tissue in cases who underwent open surgery due to ineffective LITT showed reactive gliosis and sclerosis of the vascular structures, which did not resemble the CMs common pathological characteristics (1, 4, 18).

Cyst formation in the location of ablated CM was the only reported long-term complication. The patient had CM formation after the SRS and 18 months after LITT, developed a growing cyst in previous location. Authors assume that this event could not be originated from the initial SRS (due to the duration between SRS and cyst formation), and vascular damage of LITT might be the probable cause (18).

Small sample sizes in most studies, heterogeneous CMs’ locations, the thinness of MRI slices used to evaluate volume measurements in some studies, patients lost to follow-up and also the absence of long-term follow-up, lack of high-power studies, and insufficient power to adequately prove the efficacy or safety profile of LITT for CMs are all limitations of this systematic review.

Further randomized controlled studies with larger patient sample sizes and adequate follow-up are needed to further validate the efficacy of LITT for CMs.

## Conclusion

Based on the current reports, LITT can be regarded as a treatment option for supratentorial CMs and with more caution in the deep and eloquent area’s lesions, where surgical resection is high risk and patients prefer to undergo a minimally invasive procedure as a first attempt. The majority of the reported complications are similar to those of LITT for other pathologies and were not specific to CMs. High-powered studies with a larger sample size and long-term follow-up, are needed to provide more information about the safety and efficacy of this method.

## Data Availability

The original contributions presented in the study are included in the article/Supplementary Material, further inquiries can be directed to the corresponding author/s.
